# Enhanced Nonlinear Optical Effect in Hybrid Liquid Crystal Cells Based on Photonic Crystal

**DOI:** 10.1186/s11671-017-2217-3

**Published:** 2017-07-06

**Authors:** Svitlana Bugaychuk, Andrey Iljin, Oleg Lytvynenko, Ludmila Tarakhan, Lulmila Karachevtseva

**Affiliations:** 1 Institute of Physics, National Academy of Sciences of Ukraine, Prospect Nauki 46, Kiev, 03028 Ukraine; 2 V.E. Lashkaryov Institute of Semiconductor Physics, National Academy of Sciences of Ukraine, Prospect Nauki, 43, Kiev, 03028 Ukraine

**Keywords:** Hybrid liquid crystal cell, Photonic crystal, Raman-Nath self-diffraction, Kerr-like nonlinear medium, Nonlinear susceptibility, Nonlinear refractive coefficient, 42.65.An, 81.07.Pr, 42.70.Df

## Abstract

Nonlinear-optical response of photorefractive hybrid liquid crystal (LC) cells has been studied by means of dynamic holographic technique in two-wave mixing arrangement. The LC cells include nonuniform silicon substrates comprising a micrometer-range photonic crystal. A thin LC layer is set between silicon substrate and a flat glass substrate covered by a transparent (ITO) electrode. A dynamic diffraction grating was induced in the LC volume by the two-wave mixing of laser beams with simultaneous application of DC electric field to the cell. Theoretical model of Raman-Nath self-diffraction was developed. This model allows for calculation of nonlinear optical characteristics in thin samples on the base of two-wave mixing experimental data, and with taking into account light losses on absorption and/or scattering. The hybrid LC cells demonstrate strong nonlinear optical effect, prospective for many applications in electro-optical microsystems, such as SLMs, as well as in multi-channel systems.

## Background

Among the main advantages of optical processing systems is their ability to utilize the high temporal and spatial bandwidth of photonics. A fundamental component in these systems is a device that modulates light. Spatial light modulators (SLM), imposing information onto optical data fields in optical information processing systems, for long have been considered essential for efficient exploitation of the speed, parallel-processing, and interconnection capabilities inherent to optics. These devices generally modify the phase, polarization, amplitude, and/or intensity of a spatial light distribution as a function of electrical drive information or the intensity of another light distribution [1]. The advantages of liquid crystal electro-optic materials for SLM’s include their high birefringence and low voltage operation. Placing liquid crystals on top of silicon integrated circuitry was proposed in the early 1980’s to produce handheld displays [2]. Due to the widest manufacturing of silicon integrated circuits, which may contain photodetectors, amplifiers, and memory elements, the electro-optical liquid crystal on silicon (LCoS) SLM’s became a standard tool in the majority of optical laboratories. The next and highly desired step suggests the all-optical information handling, which could be achieved via light-controlled modification of the optical properties of the medium. Many of optically addressed SLM’s adopted the basic sandwich structure with the photoconductor transferring a bias voltage applied to the sandwich to a modulating material, e.g., liquid crystals in the liquid crystal light valve (LCLV) [3].

Almost all existing nonlinear optical effects have been observed in dye-doped LC compositions, in which the absorbing dye molecules trigger the LC director reorientation [4]. Alternatively, light action on the photosensitive molecules affects the order parameter of LC that, in turn, results in substantial and fast changes of the local refractive indices of the LC [5]. Last year, the popular photo-sensitive centers become nanoparticles embedded in the LC volume [6]. Not absorbing LC systems have also attracted a lot of interest as photorefractive pure-nematic-LC light valves. The main nonlinear optical mechanism in LC is collective reorientation of molecules in a bulk of LC under laser excitation that often appears with applied electric field. In the present work, we use pure nematic LC. The nonlinear optical mechanism in such as LC cells suggests a surface-induced photorefractive effect, which changes the orientation of the LC molecules in the bulk initiated from the surface. This effect was investigated in LC cells containing different surface materials, such as photorefractive polymer layers [7, 8], conductive layers with embedded impurities [9], noble metal plates [10], and photorefractive crystals [11]. As a rule, the initial orientation of the molecules on a surface was the planar. Another kind of cells, which exhibit surface-induced photorefractive effect, consist of simple glass substrates covered with ITO electrodes and filled with a pure nematic LC, but the studied main feature is the homeotropic orientation of molecules [12, 13]. Such effect is studied in the present work. We use, however, hybrid cells where one of the substrates is a photonic crystal made from silicon. Enhancement of physical properties of materials deposited in nano- or microstructured surfaces, including optical, electrical, and other properties becomes one of a priority direction of the fundamental nano-science. In our research, we investigate the possibility of strengthening of nonlinear optical effect in an LC cell which is due to the influence of a microstructured surface of a substrate, which forms the cell.

To investigate the nonlinear optical properties in hybrid LC cells containing a reflective surface, we apply the dynamic holographic technique based on the two-wave mixing of laser beams in the reflection geometry [14]. It is known that the dynamic holography is based on three main effects, which act simultaneously: (1) creation of periodic interference pattern inside a nonlinear medium with the help of two or more coherent laser beams; (2) modulation of the refractive index under action of this interference pattern; in our case, it means inducing a phase dynamic grating inside a nonlinear medium; (3) self-diffraction of the recording beams on the dynamic grating. So far, the wave-mixing is known as an effective technique for many applications in nonlinear optics (see, for example, [15]). Suffice it to mention spatial multiplexing and switching of laser beams, all-optical control of parameters of the beams, dynamic optical memory, logic, etc. In the present work, we demonstrate that this method can also be applied as a simple experimental technique to determine nonlinear optical characteristics of thin films. We have developed a mathematical approach to calculate the coefficient of nonlinear refraction in a Kerr-like medium, from which the nonlinear optical susceptibility can be determined. The mathematical model covers the self-diffraction of waves in the Raman-Nath regime, i.e., for the condition of a thin grating. This approach generally is satisfied for the majority of thin samples that typically have thickness of up to tens of micrometers. Such media include also LC cells. Note that an alternative method to determine the optical nonlinear susceptibility of transparent materials is the z-scan technique [16]. But we show that the two-wave mixing method is rather simple for the experimental realization and is very suitable for the investigation of the dynamical media, including that that works only in the reflection geometry.

## Methods

### Materials and Samples

**Fig. 1:**
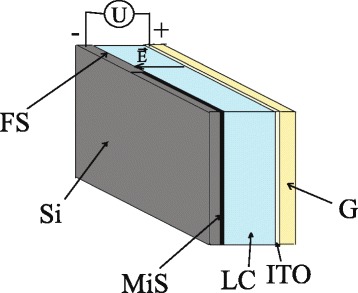
Structure of a hybrid LC cell: silicon substrate (*Si*); microstructured silicon surface (*MiS*); flat silicon surface (*FS*); liquid crystals (*LC*); glass substrate (*G*); ITO electrode (*ITO*); applied voltage (*U*); electric field vector ($\overrightarrow{E}$)

Structure of the hybrid LC cells is shown in Fig. 1. The hybrid cell has a sandwich-like type, is formed by glass and silicon substrates and filled with nematic LC; its edges are glued. The thickness of the nematic LC layer is 20 μm. The flat glass substrate is covered with ITO electrode. The second substrate is cut from a phosphorus-doped silicon wafer, its dimensions are 17 × 17mm^2^. Its resistivity is 4.5 Ω⋅cm, its thickness is 380 μm, and its orientation is 〈100〉. The silicon substrate contains two areas, namely, a surface with periodic microstructured part, which is actually a photonic crystal in the micrometer range, and a flat part of the surface.

**Fig. 2:**
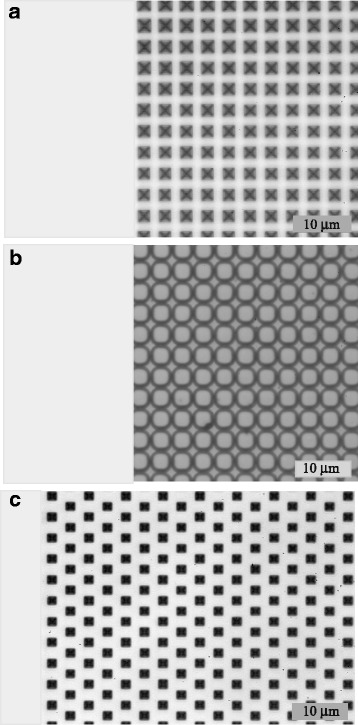
Images of microstructured silicon surface made in optical microscope. Micropyramids have the following shapes: regular pyramids (1), pits (2) and slightly truncated pyramids (3)

The three Si substrates, used in our investigation, are shown in Fig. 2. The microstructures represent etched pits arranged in a square matrix (the substrates 1 and 2) or in a triangular matrix (the substrate 3). The pits are square micropyramids, which differ by the shape, size, and arrangement. There are (1) regular pyramids, (2) truncated pyramids (pits), and (3) slightly truncated pyramids. Regular micropyramids are 2 μm in height, the same base’s side of 2 μm, and periodicity of 3 μm. Pits have similar parameters, but they are close packed. Slightly truncated pyramids have the base side 1.5 μm and periodicity 3.5 μm. The microstructures were formed on the polished side of the silicon wafer by standard photolithographic technique followed by anisotropic etching for the substrates 1 and 3, or by plasma etching for the substrate 2.

Two nematic LC were used: pure nematic 5CB (4×-(*n*-pentyl)-4-cyanobiphenyl) and nematic mixture E7. In all cases, molecular orientation in a liquid crystal layer is homeotropic and it appears spontaneously at ITO and silicon surfaces with processing temperature being kept not higher then 50 °C. We have investigated four samples, which differ by the shape of microstructure and by the used LC. The samples with silicon substrates 1 and 2 consist of two parts: one part contains a miscrostructured surface, and the second part is a flat. In such samples we have the opportunity to compare the nonlinear response in a flat cell (containing a flat part of the Si substrate), and in a microstructured cell (containing a microstructured part of the Si substrate).

This way, in our experiments we have the following hybrid cells:

1. abbreviated M1: glass substrate + ITO/5CB/Si substrate 1, microstructured surface
2. abbreviated F1: glass substrate + ITO/5CB/Si substrate 1, flat surface
3. abbreviated M2: glass substrate + ITO/5CB/Si substrate 2, microstructured surface
4. abbreviated F2: glass substrate + ITO/5CB/Si substrate 2, flat surface
5. abbreviated M3: glass substrate + ITO/5CB/Si substrate 3, microstructured surface
6. abbreviated M4: glass substrate + ITO/E7/Si substrate 3, microstructured surface

### Experimental Set-up

**Fig. 3:**
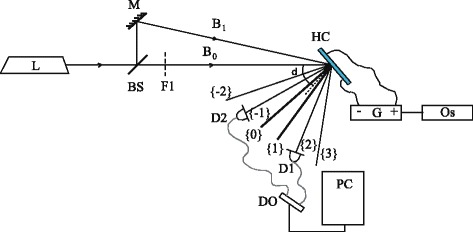
Scheme of experimental set-up: laser (*L*); mirror (*M*); beam splitter (*BS*); optical filter (*F1*); beams (*B*_*0*_) and (*B*_*1*_); photodiodes (*D1*) and (*D2*); hybrid LC cell (*HC*); oscilloscope (*Os*); generator (*G*); digital oscillographe (*DO*); computer (*PC*). The output diffraction orders are {0} and {1} the main orders; {−1} and {2} the first diffraction orders; {−2} and {3} the second diffraction orders. *δ* is the rotation angle of the cell

Figure 3 shows the scheme of the experimental set-up, which is based on the dynamic holographic method with two-wave mixing. Continuous semiconductor Nd:YAG laser (frequency doubling, *λ* = 532 nm, *P* = 52 mW, single-mode generation) is a light source. By means of a beam splitter BS and a mirror M, the laser radiation is split into two beams *B*_0_ and *B*_1_, converging on the cell at a small angle *θ* ≈ 0.01 rad. The input intensities *I*_0_ and *I*_1_ are equalized with the help of the filter F1, and in our case, *I*_0_ = *I*_1_ = 3.3 W/cm^2^. A laser spot diameter on a cell is 1 mm.

Two laser beams *B*_0_ and *B*_1_ form an interference pattern inside the sample. Both incident beams have linear *p* polarization. So the intensity modulation interference pattern is created. The normal LC cell makes the angle *δ* with the bisector of *B*_0_ and *B*_1_. The DC electric field is applied due to the source *G* with the voltage *U* controlled in the range from 0 to 15 V. The ITO glass substrate is set as the positive electrode. We measured intensities of the first diffraction orders {−1} and {2} by using photodiodes D1 and D2 via computer-controlled digital oscilloscope DO. The angles of cell rotation were adjusted to maximize the diffraction signal and appeared to be *δ* ≈ 40 − 55^0^ for different samples. These results coincide with the experiments of other groups (for example [9, 12]); the reason for such an effect is beyond the scope of our study.

### Model of Self-diffraction of Waves in Raman-Nath Approximation

Self-diffraction of waves has been considered in several works [17, 18]. When the self-diffraction of two input waves on the photoinduced thin refractive-index grating takes place, many diffraction orders appear at the output. By measuring the intensities in the first diffraction orders, one can calculate the modulation depth of the grating (*Δn*). Since in the Kerr-like medium the condition *Δn* = *n*_2_ *I*_0_ is valid (where *I*_0_ is the intensity of the exciting beam), the coefficient of the nonlinear refraction *n*_2_ is possible to calculate.

**Fig. 4:**
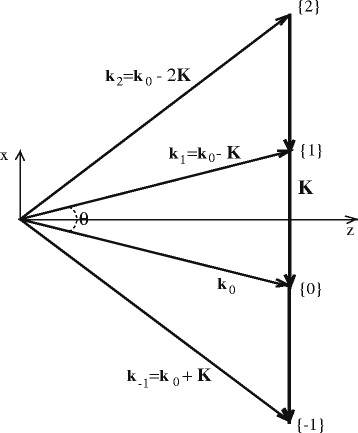
Wave-vector diagram of self-diffraction of two coherent waves (described by ${\overrightarrow{k}}_0$ and ${\overrightarrow{k}}_1$) in the Raman-Nath approximation

In this section, we look for the solution for the diffraction efficiency of the first diffraction orders in the case of not-shifted sinusoidal refractive index grating. Then intensities in symmetrical orders will be equal. The modeling is started from the wave equation, in which both the electric field $\overrightarrow{E}$ and the modulated part of the dielectric permittivity *Δε* resulting from the Kerr-like nonlinear effect depend on the coordinate *z* (along the wave propagation):

1 $$ {\nabla}^2\overrightarrow{E}\left( z, t\right)=\frac{1}{c^2}\frac{\partial^2}{\partial {t}^2}\left[{\varepsilon}_0+\varDelta \varepsilon \left( z, t\right)\right]\overrightarrow{E}\left( z, t\right) $$

where *c* is the light velocity in the vacuum, ${\varepsilon}_0={n}_0^2$ represents the dielectric permittivity of a medium and *n*_0_ is its refractive index. We will look for the solution of the wave equation (1) in the sum of all diffraction orders, which are plane waves polarized in the direction of the axis $\overrightarrow{y}$:

2 $$ \overrightarrow{E}=\frac{1}{2}\overrightarrow{y}\left\{{\displaystyle \sum_{m=-\infty}^{+\infty }{\overrightarrow{A}}_m\left( z, t\right){e}^{i\left[{\omega}_0 t-\left({\overrightarrow{k}}_0- m\overrightarrow{K}\right)\overrightarrow{r}\right]}{e}^{-\frac{1}{2}\alpha z}+ c. c.}\right\} $$

where *ω*_0_ is the frequency of the laser radiation, $\overrightarrow{r}$ is the coordinate, and “*c. c*.” denotes the complex conjugate term. In our representation, it is convenient to express the attenuation coefficient as *α* = *α*_abs_ + *α*_sc_, which takes into account the losses of laser radiation both on absorption *α*_abs_ and scattering *α*_*s*_. Figure 4 shows the wave-vector diagram of self-diffraction in Raman-Nath approximation. It demonstrates that the wave of the *m*-th diffraction order corresponds to the spatial direction described by the wave-vector ${\overrightarrow{k}}_m$. Diffraction orders *m* = 0 and *m* = 1 belong to two exciting beams *B*_0_ and *B*_1_. The wave-vector of the *m*-th diffraction order is ${\overrightarrow{k}}_m={\overrightarrow{k}}_0- m\overrightarrow{K}$, and $\overrightarrow{K}$ is the wave-vector of the photoinduced grating. The permittivity modulation *Δε* is defined in the form of a grating:

3 $$ \varDelta \varepsilon \left( z, t\right)=\frac{1}{2}\left[{\overrightarrow{\varepsilon}}_1\left( z, t\right){e}^{- i\overrightarrow{K}\overrightarrow{r}}+ c. c.\right] $$

By substituting solution (2) and (3) into Eq. (1), one obtains the relation for a slow variable amplitude *A*_*m*_ of the *m*-th order:

4 $$ \frac{\partial {A}_m}{\partial z}+ i\frac{\left({k}_0^2-{k}_m^2\right)}{2{k}_{m z}}{A}_m-\frac{1}{2}\alpha \cdot {A}_m=- i\frac{k_{\upsilon}^2}{4{k}_{m z}}\left[{\varepsilon}_1{A}_{m+1}+{\varepsilon}_1^{*}{A}_{m-1}\right] $$

where *k*_*υ*_ = 2*π*/*λ* is the wave-vector in the vacuum, and the mark “∗” denotes the complex conjugation. Since in our representation, the main recording beams of the orders *m* = 0 and *m* = 1 are identical at the input, as well as throughout the thickness of the sample, it follows that *ε*_1_ is a real and does not depend on the coordinate *z* (see [18]): ${\varepsilon}_1(t)={\varepsilon}_1^{*}(t)$. For further transformation of Eq. (4), we introduce a new function ${U}_m\left( z, t\right)={A}_m\left( z, t\right) \exp \left(-\frac{1}{2}\alpha z\right) \exp \left( im\frac{\pi}{2}\right)$, designate *T* = exp(−*αz*) the losses of the light intensity in the medium, and define ${\varepsilon}_1= T{\tilde{\varepsilon}}_1$. By introducing a new variable $\tilde{z}={k}_{\upsilon}/\left({n}_0 \cos \left(\theta /2\right)\right)\cdot \left(1- T\right)/\left(2\alpha \right)$, where *θ* is the converging angle, Eq. (4) may be written as:

5 $$ 2\frac{\partial {U}_m}{\partial \tilde{z}}={\tilde{\varepsilon}}_1\left[-{U}_{m+1}+{U}_{m-1}- i\frac{2 m\left( m-1\right)}{\phi}{U}_m\right] $$

where parameter *ϕ* is defined by $\phi = T{\tilde{\varepsilon}}_1/\left(2{n}_0^2{ \sin}^2\left(\theta /2\right)\right)$.

In the conditions of the Raman-Nath approximation, it is possible to neglect the last term on the right part of the Eq. (5) (Ref. [18]), i.e., 2*m*(*m* − 1)/*ϕ* < < 1 for any *m*. Then by introducing a new variable $\zeta =\tilde{z}{\tilde{\varepsilon}}_1(t)$, we obtain our main equation in the Raman-Nath approximation:

6 $$ 2\frac{\partial {U}_m\left(\zeta, t\right)}{\partial \zeta}+{U}_{m+1}\left(\zeta, t\right)-{U}_{m-1}\left(\zeta, t\right)=0 $$

This relation formally is a well-known representation for the Bessel function, so its solution may be expressed by means of Bessel functions in the form:

7 $$ {U}_m\left(\zeta, t\right)={\displaystyle \sum_{n=0}^{\infty }{C}_n^m(t){J}_n\left(\zeta \right)} $$

The Eq. (6) satisfies properties of symmetry ${U}_0{U}_0^{*}={U}_1{U}_1^{*}$ for the pair of the main beams, as well as for all pairs of the diffracted beams (${U}_2{U}_2^{*}={U}_{-1}{U}_{-1}^{*}$). Note that at *z* = 0, *E*_0_(0, *t*) = *E*_1_(0, *t*) ≠ 0, but *E*_*m*_(0, *t*) = 0 for *m* ≠ 0, 1, and then output intensities of the first diffraction orders *I*_{−1}_(*d*, *t*) and *I*_{2}_(*d*, *t*) will be equal and defined by a formula (see also [18]):

8 $$ {I}_{\left\{-1\right\}}\left( d, t\right)={I}_{\left\{2\right\}}\left( d, t\right)= T{I}_0\left(0, t\right)\left[{J}_1^2\left(\zeta \right)+{J}_2^2\left(\zeta \right)\right] $$

where *d* is the thickness of a nonlinear medium; *I*_0_ is the intensity of a laser beam; *J*_1_ and *J*_2_ are the Bessel functions of the first kind of the first and second orders, respectively. Since in our case, the intensities *I*_0_ and *I*_1_ are equal, i.e., 2*I*_0_ = *I*_laser_, consequently, the value of *ζ* can be written as:

9 $$ \zeta =\tilde{z}{\tilde{\varepsilon}}_1=\frac{k_{\upsilon}}{n_0\cdot \cos \left(\theta /2\right)}\frac{1- T}{2\alpha}{\tilde{\varepsilon}}_1\approx \frac{k_0}{n_0}\frac{1- T}{2\alpha}2{n}_0\varDelta n $$

In a Kerr-like medium *Δn* = *n*_2_ *I*_0_, where *n*_2_ represents the nonlinear refraction coefficient, then the final value of *ζ* has a more simple form:

10 $$ \zeta =\frac{2\pi}{\lambda}\frac{1- T}{\alpha}{n}_2{I}_0 $$

The diffraction efficiency *η* of the first diffraction order is determined as *η* = *I*_{−1}_/(*TI*_0_). On the other hand, the diffraction efficiency may be obtained experimentally by measuring the intensity *I*_{−1}_ and the transmission coefficient of a cell *T*. The common formula (8) is valid to calculate the diffraction efficiency in a large range. For smaller diffraction efficiencies, *η* ≤ 2%, the good approximation will be only the first polynomial term of (8): *η* ≈ *ζ* ^2^/4. Consequently, one can obtain a simple relation for *n*_2_:

11 $$ {n}_2=\frac{\lambda}{\pi}\frac{\alpha}{1- T}\frac{\sqrt{\eta}}{I_0} $$

It should be noted that the derived Eq. (11) has the same form, as the equation which is conventionally obtained for the case of the diffraction of only one probe beam from a given refractive index grating (see, for example, Ref. [19]). But for large values of *η*, that is usual for LC cells, a more precise relations (8) and (10) should be used to calculate the values *ζ* and *n*_2_.

Knowing the value *n*_2_, one can define the nonlinear susceptibility of the medium with the help of the expression:

12 $$ {\chi}^{(3)}\left[\mathrm{esu}\right]={n}_2\left[\frac{\mathrm{c}{\mathrm{m}}^2}{W}\right]\cdot \frac{9\cdot {10}^4}{4\pi} c\cdot {\varepsilon}_e\cdot {n}_0^2 $$

where *ε*_*e*_ is the electric constant. In the case of nematic 5CB, we use the refractive index for the homeotropic orientation of molecules *n*_0_ = *n*_⊥_ = 1.51 as that of the ordinary refractive index; similarly for nematic mixture E7, *n*_0_ = *n*_⊥_ = 1.5268.

The developed theoretical approach is valid for thin films possessing Kerr-like optical nonlinearity, when the light losses on the both the absorption and scattering are large. Since the self-diffraction method does not require an additional laser source as a probe, the two-wave mixing becomes a simple method for diagnostics of optical nonlinearity of thin mediums including LC cells.

## Results and Discussions

We suggest the nonlinear optical mechanism in hybrid LC cells to be a surface-induced photorefractive effect, which changes the orientation of the LC molecules in the bulk initiated from the surface [12, 13]. In the two-wave mixing experiments, the sample is illuminated by a periodic light interference pattern formed by two coherent laser beams. This pattern creates a spatial charge modulation on the LC-substrate interface. Resulting periodic distribution of the electric field on the surface stimulates modulation of the director orientation on the substrate. The reorientation of the molecules starts at the surface and spreads into LC volume.

**Fig. 5:**
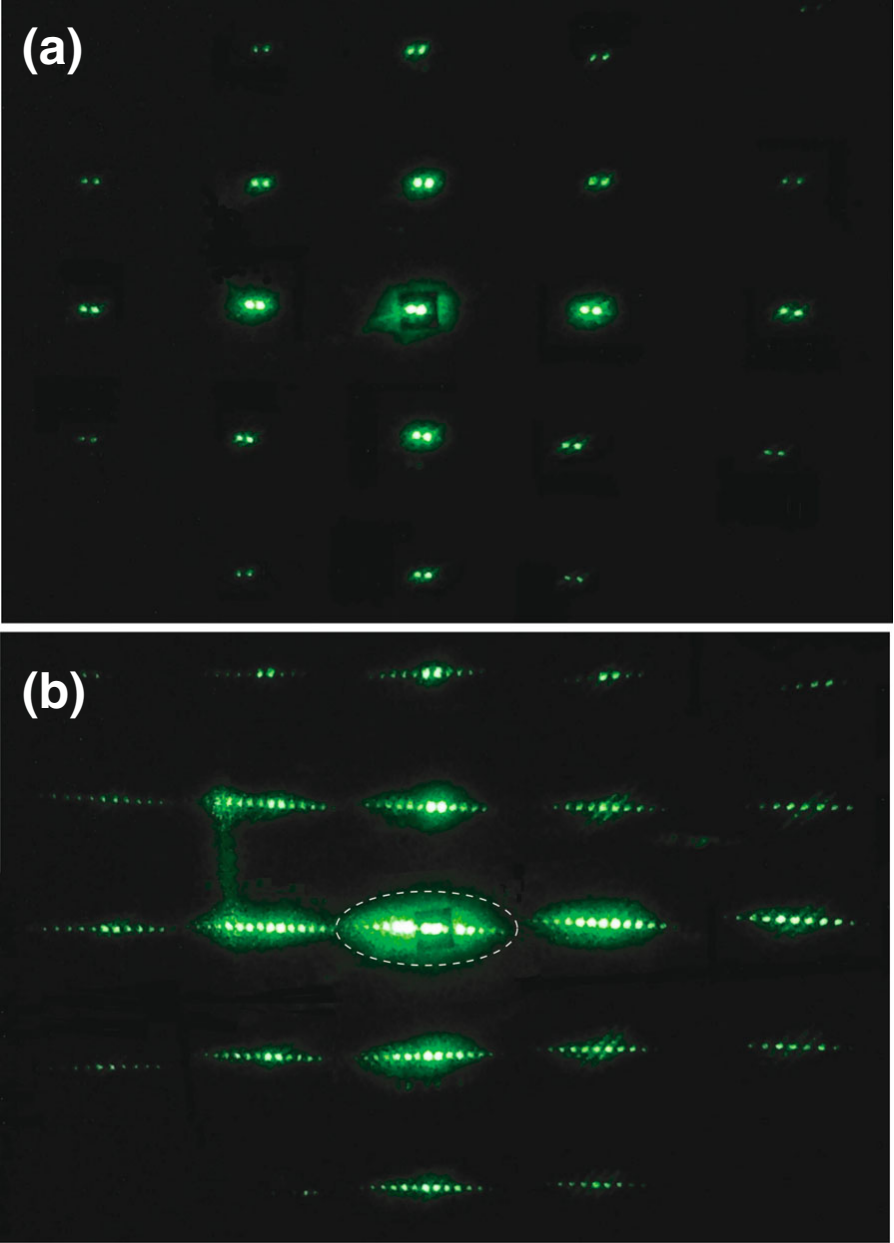
Typical patterns of scattering for two interfering laser beams formed by hybrid nonlinear LC cell with microstructured surface. **a** Scattering pattern of a hybrid cell without applied electric field. **b** Formation of many diffraction orders (the Raman-Nath self-diffraction) at application of DC electric voltage. The centre line is shown in (**b**) by a *dash line*

Typical two-wave mixing experimental performance with hybrid LC cells is shown in Fig. 5. In the absence of electric field, we observe a regular two-dimensional structure of the main laser beams reflected from a microstructured substrate of the cell (Fig. 5a). After application of DC voltage, many diffraction orders appear aside every pair of the main beams due to excitation of refractive index grating inside the cell (Fig. 5b). In the case of flat cells F1 and F2, there is no multi-scattering pattern, we observe only one center line after applying the voltage. We measured the intensities in the first diffraction orders {−1} and {+2} in the centre line in the steady state for all cells: flat cells and cells with microstructured surface. Then we calculated the diffraction efficiency according to *η* = *Ī*_{−1}_/(*TI*_0_), where *Ī*_{−1}_ is the average intensity of two first diffraction orders (*Ī*_{−1}_ = (*I*_{−1}_ + *I*_{2}_)/2) and *T* is the transmission coefficient of the cell.

Note, that the surface-induced photorefractive effect, exploited in our experiments, depends strongly on the rotation angle of the sample relative to the bisector of converge angle between two input waves (see e.g., [9, 12, 13]). Thus, with the normal position of the sample, when the plate of the sample is perpendicular to the waves’ bisector, no diffraction orders are observed. At the same time in our case, when the sample is rotated relative to this bisector, the photoinduced refractive index grating appears to be shifted relative to the light interference pattern. This effect should be manifested in the existing of energy transfer between diffraction orders. In the case of our hybrid cells and two laser beams with equal input intensities, we have observed that the difference of the intensities in the first diffraction orders does not exceed 10%. We have taken an average value between these two measured intensities that is used for further calculations of the coefficient of the nonlinear refraction. This value belongs to the accuracy range of our estimations of the nonlinear optical coefficients. Also note that the developed mathematical model is reduced to rather simple resulting formula and does not include the changes of wave phases in a volume of a nonlinear layer. The effect of nonlocal response in the medium and the energy transfer between waves will be considered carefully in our next works.

In Table 1, we gather experimental parameters measured for the hybrid LC cells. The transmission coefficient is defined as *T* = *I* ^out^/*I*_0_, where *I*_0_ is the intensity of a single incident beam, and *I* ^out^ is the intensity of the output beam. *T* includes two parts: *T* = *R*_*s*_ *T*_*a*_, where *R*_*s*_ takes into account the intensity loss for scattering from the microstructured surface to form a periodic light pattern; and *T*_*a*_ = exp(−*αd*_eff_) describes intensity loss on absorption during light propagation in a bulk of LC cell. In Table 1 also we present the values *d*_eff_, which is an effective thickness for a propagating beam in a cell. Note, in our measurements we neglect the losses on light reflection from input glass boundary of a cell.

**Table 1:** Experimental measurements of light losses in hybrid cells and effective thickness *d*_eff_ of the cells

Hybrid LC cell	*T*	*R*_*s*_	*T*_*a*_	*δ*, ^0^	*d*_eff_, μm
M1	0.09	0.104	0.865	42	59.79
F1	0.14	0.234	0.584	42	59.79
M2	0.0436	0.054	0.807	50	52.22
F2	0.134	0.358	0.374	50	52.22
M3	0.145	0.25	0.58	40	62.22
M4	0.17	0.25	0.68	55	48.83

*T* is total transmission coefficient of a cell; *R*_*s*_ is the reflection coefficient of a pure silicon surface; *T*_*a*_ is the transmission coefficient which includes light absorption in LC layer; *δ* is the rotation angle of the cell relative to normal incidence of light

**Fig. 6:**
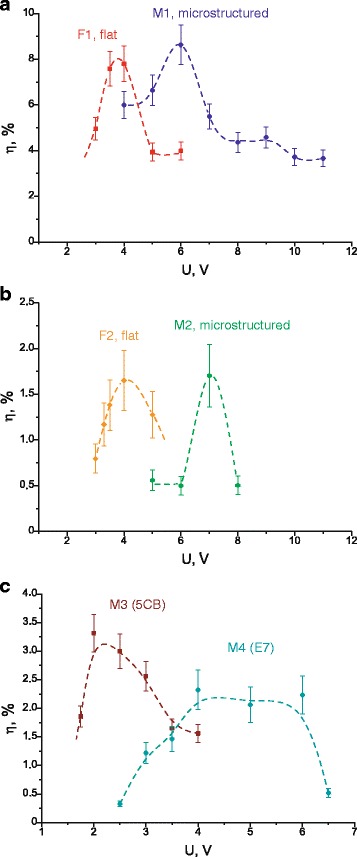
Diffraction efficiency of first diffraction order in dependence on applied voltage. **a** Cells M1 and F1. **b** Cells M2 and F2. **c** Cells M3 and M4. *Dashed lines* presented for the visualisation only

Measured diffraction efficiencies for all cells in dependence on applied voltage are shown in Fig. 6. One can see that the diffraction efficiency reaches its maximum for a certain voltage, which differs in various cells; this voltage is higher for microstructured cells in comparison with flat one (compare F1 and M1, F2 and M2); as well as this voltage is changed in dependence on a shape of the microstructure (compare M1, M2, M3, M4). We also emphasize that the diffraction efficiencies reach rather large values in LC cells (up to 9% for the cells M1 and F1). We use measured values of *η* to calculate nonlinear optical characteristics of investigated LC cells, namely, the coefficient of nonlinear refraction *n*_2_ and effective nonlinear susceptibility *χ* ^(3)^, taking into account that the LC cells possess Kerr-like optical nonlinearity.

**Fig. 7:**
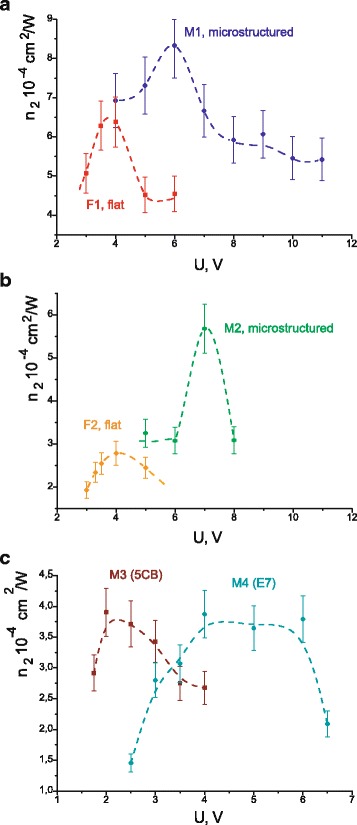
Calculated coefficients of nonlinear refraction in dependence on applied voltage for cells M1 and F1 (**a**); M2 and F2 (**b**); M3 and M4 (**c**). *Dashed lines* are for the visualisation only

Coefficients of nonlinear refraction calculated from experimental measurements of the diffraction efficiencies are shown in Fig. 7 for all cells. In the case of cells M1 and F1 as well as M3 and M4, *n*_2_ is calculated from the main formulas (8) and (10) as the measured diffraction efficiencies *η* > 3.5%. We use the approximate formula (12) for the cells M2 and F2, as the obtained *η* < 2%. We obtain that the maximal coefficient of nonlinear refraction is higher in a cell with microstructured substrate as compared with flat cells (see Fig. 7a, b). Table 2 presents the values of nonlinear susceptibility calculated from the maximal values of *n*_2_ in Fig. 7. Nonlinear susceptibility appeared to be essentially enhanced (by 30–100%) in the cells with microstructured substrate with respect to the cells made of flat substrates. Reasons leading to increasing modulation depth of the dynamical grating in the cells contained microstructured substrates are the subject for further research. We suppose this effect to be connected with initial reorientation of molecules arising on microstructured surface.

**Table 2:** Maximum values of nonlinear susceptibility of investigated hybrid LC cells

Nonlinear LC cell	*χ* ^(3)^ 10^−2^ [esu]	% of enhancement
M1	3.6 ± 0.7	30% (between M1 and F1)
F1	2.8 ± 0.6	
M2	2.5 ± 0.5	100% (between M2 and F2)
F2	1.2 ± 0.2	
M3	1.5 ± 0.3	
M4	1.7 ± 0.4	

## Conclusions

We have investigated the nonlinear optical effect in novel hybrid LC cells based on a silicon photonic crystal. The cell consists of two different materials separated by a thin LC layer with homeotropic orientation of molecules. One material is a glass substrate with ITO electrode. The second one is silicon substrate with periodic microstructured surface. Microstructures in a shape of periodically arranged micro-pyramids are etched on the silicon surface by applying the chemical photolithography method or plasma etching one.

We apply the dynamic holography method with two-wave mixing to define the efficiency of self-diffraction of the dynamic grating induced in LC layer. A theoretical model for the Raman-Nath self-diffraction, offered for calculating the diffraction efficiency in the first diffraction orders, have allowed us to determine the nonlinear refraction coefficient *n*_2_, and nonlinear susceptilibity *χ* ^(3)^ of the cells. We have also made a comparative analyses of nonlinear parameters obtained for cells with and without microstructures. Nonlinear susceptibility appeared to be essentially enhanced (by 30 − 100%) in the microstructured cells with respect to the cells made of flat surfaces. The underlying mechanism of the optical nonlinearity is the surface-induced photorefractive effect in the pure nematic LC. The increased modulation depth of the refractive index might be connected with initial reorientation of the molecules arising on the microstructured substrate.

The developed theoretical approach could be valid for determination of nonlinear optical characteristics of thin films possessing Kerr-like optical nonlinearity, in which the losses on the both absorption and scattering are large, as well as in the either transmission or reflection geometries. Photorefractive hybrid LC cells are perspective as new samples of electro-optical microsystems, including multi-channel SLMs. Additionally, two-wave mixing technique in such nonlinear cells may be successfully implemented in multi-channel couplers, switches, and optical communication lines. They may be also applied in networks, if to ensure the independent control of each channel in LCD structures.

## Abbreviations

5CB: 4′-(*n*-pentyl)-4-cyanobiphenyl
E7: Liquid crystal mixture
F1: Hybrid LC cell, composed by flat part of Si plate 1/5CB/glass plate covered by ITO
F2: Hybrid LC cell, composed by flat part of Si plate 2/5CB/glass plate covered by ITO
ITO: Conductive layer of indium-tin-oxide
LC: Liquid crystals
M1: Hybrid LC cell, composed by microstructured part of Si plate 1/5CB/glass plate covered by ITO
M2: Hybrid LC cell, composed by microstructured part of Si plate 2/5CB/glass plate covered by ITO
M3: Hybrid LC cell, composed by microstructured part of Si plate 3/5CB/glass plate covered by ITO
M4: Hybrid LC cell, composed by microstructured part of Si plate 4/E7/glass plate covered by ITO
Si: Silicon
